# The antiepileptic potential of *Vateria indica* Linn in experimental animal models: Effect on brain GABA levels and molecular mechanisms

**DOI:** 10.1016/j.sjbs.2022.02.059

**Published:** 2022-03-04

**Authors:** Ali Mohamed Alshabi, Ibrahim Ahmed Shaikh, Syed Mohammed Basheeruddin Asdaq

**Affiliations:** a Department of Clinical Pharmacy, College of Pharmacy, Najran University, Najran, Saudi Arabia; b Department of Pharmacology, College of Pharmacy, Najran University, Najran, Saudi Arabia; c Department of Pharmacy Practice, College of Pharmacy, AlMaarefa University, P.O Box 71666, Dariyah, 13713 Riyadh, Saudi Arabia

**Keywords:** Epilepsy, *Vateria indica*, Antioxidant, GABA, Antiepileptic

## Abstract

**Background:**

Epilepsy is a neurodegenerative condition characterized by uncontrollable convulsions caused by a misalignment of the central nervous system's inhibitory and excitatory branches. Vateria indica is a medicinal herb with anti-inflammatory, anthelmintic, antiulcer, antitumor, and anticancer properties.

**Objectives:**

To investigate the antiepileptic activity of *Vateria indica* using maximal electrical shock (MES), pentylenetetrazole (PTZ), and isoniazid (INH) induced experimental animal models.

**Methodology:**

*Vateria indica* bark was subjected to Soxhlet extraction using ethanol and quantitative and qualitative analysis was performed. The antiepileptic activity of *Vateria indica* bark extract (VIE) was investigated using different animal models in mice. GABA levels in the brain and antioxidant capacity *in vitro* were estimated.

**Results:**

Treatment of mice with VIE significantly reversed the MES-induced convulsions, which was reflected by the decrease in the duration (sec) of all the phases of MES-induced convulsions, with an increment in the GABA levels. In the PTZ and INH models, pretreatment with VIE delayed the latency to clonic convulsions (*p* 0.001), reduced the intensity and duration of clonic convulsions, and reduced the mortality rate in the treatment groups in a dose-dependent manner. VIE intervention dose-dependently restored brain GABA levels. VIE also exhibited significant *in-vitro* antioxidant activity.

**Conclusion:**

Overall, the findings imply that *Vateria indica* has substantial antiepileptic activities, mediated by positive GABAergic neurotransmission and antioxidant capabilities. To summarize, *Vateria indica* may provide adequate protection against epileptic seizures, suggesting that it could be used to treat petitmal and grandmal epilepsy. We plan to provide pure lead compounds derived from *Vateria indica* in the future in order to better understand the role it could play in the development of natural anticonvulsant drugs.

## 1 Introduction

Epilepsy is a prevalent disease marked by spontaneous seizures that occur due to an asymmetry amid inhibitory and excitatory branches of the CNS—created by an elevation in glutamatergic and a decline in GABAergic activity (Meldrum, 1999). It is predicted that 50 million humans worldwide have epilepsy, with low-income countries accounting for more than 85 percent of cases.

The most common focal seizures are associated with Temporal-lobe epilepsy (TLE), which has been linked to approximately 60% of all epilepsy patients. TLE is related to hippocampal sclerosis and is often resistant to conventional medicines. Management of TLE is complex, and often, surgery is the last option (French et al., 1993).

In Saudi Arabia, epilepsy is one of the most prevalent neurological disorders, with a rate of 6.54 per 1000. According to a previous study, the majority (28%) have partial seizures, followed by generalized seizures (21%), and 3.55 /1000 children under six years of age had febrile convulsions (Al Rajeh et al., 2001).

Conventional antiepileptic medicines (AEMs) are used in the current epilepsy treatment regimen. However, AEMs are known to produce congenital anomalies and other long-term adverse effects, including weight gain, hepatotoxicity, colitis, nephrolithiasis, metabolic acidosis, paresthesia, glaucoma, agranulocytosis, and neurological symptoms (Walia et al., 2004). With the existing AEMs, about one-third of the people with epilepsy do not have complete control over seizures (Pithadia et al., 2013). As a result, there is a pressing need to discover and test new antiepileptic alternatives that can effectively tackle the actual molecular events of epilepsy while avoiding significant side effects. Plants were used as a treatment source for various diseases all over the world during the prehistoric era. The history of nutraceuticals can be traced back to alternative medicine, including phytotherapy, apothecary, herbalism, and ethnopharmacology (Makkar et al., 2020). There are a plethora of undiscovered natural products and nutrients with beneficial biological properties. Nutraceuticals have been shown to have neuroprotective effects in the treatment of neurodegenerative and psychotic disorders (van der Burg et al., 2021).

Several animal models have been developed for the screening of antiepileptic drugs. Status Epilepticus and kindling are mainly used to study TLE. The key advantage of these two models is that they provide a persistent induction of epileptic-like conditions. Generally, repeated electrical stimulations of low intensity induce focal seizure discharge, leading to a highly reliable and progressive increase in epileptic response (French et al., 1993, Abdel-Wahab et al., 2015, Lopes et al., 2015). Nevertheless, the employment of synthetic inducing agents, such as pentylenetetrazole, is equally effective for kindling induction in animal models (Corda et al., 1990).

A high dose of acute administration of pilocarpine in rats and mice is extensively utilized to investigate seizures' pathophysiology (Lopes et al., 2015, Turski et al., 1983). This model is used to study seizures' behavioural and electroencephalographic characteristics, which mimic the TLE. Similarly, Kainic acid is used to produce similar status epilepticus or TLE state in various species through administration by either an intrahippocampal, systemic, or intra-amygdaloid route (Lévesque and Avoli, 2013).

There is sufficient experimental evidence implicating brain neurotransmitters and oxidative stress in the aetiology of epilepsy. The significance of GABA in the pathophysiology and management of epilepsy has been well established. GABA counterbalances neuronal excitability in the cerebral cortex and sustains an inhibitory tone, and any imbalance can precipitate epileptic seizures (Meldrum, 1999).

It is undeniable that the botanical kingdom is a rich source of bioactive chemicals (Magaji et al., 2012). Contemporary scientific research is focused on isolating, identifying, and characterization of the bioactive components of medicinal plants that could be used as “lead” molecules in therapeutic drug discovery and development (Quintans et al., 2014). Flavonoids, which are structurally related to benzodiazepines, exert an antiepileptic effect by altering the GABAA-Cl-channel complex (Choudhary et al., 2011).

One such plant rich in flavonoids and polyphenols is *Vateria indica* Linn, also known as White Dammar, a tropical tree grown in East-Asian countries. This tree is leathery, has glabrous leaves, and can reach up to 40 m in height. It has been used as a remedial agent for ages, and it possesses anthelmintic, anti-inflammatory, antiulcer, and anticancer properties. The bark extract is abundant in flavonoids and polyphenols, rendering it a potential neuroprotective agent, as polyphenols are known as neuroprotective (Spagnuolo et al., 2016).

Previously, our research team has evaluated the protective effect of *V. indica* on memory retention and consolidation (Alshabi et al., 2020). Our results revealed that pretreatment with *V. indica* improved cognition and conferred neuroprotection in young amnesic mice. The promising results urged us to further investigate the *V. indica* ethanolic extract for antiepileptic activity through modulation of brain GABA levels and oxidative stress in mice.

The current study evaluated *Vateria indica* (VI) for its antiepileptic potential in MES and Isoniazid induced seizures as well as Pentylenetetrazole (PTZ) kindling models of TLE. There is no experimental data available in the literature for *V. indica* bark extract's antiepileptic activity. Hence, we thought it worthwhile to perform systemic research to evaluate *V. indica* bark for its antiepileptic and antioxidant potential.

## 2 Methodology

### 2.1 Research design

This study was conducted in the Pharmacology Department, Faculty of Pharmacy, Najran University, KSA, from May to June 2021.

### 2.2 Animals

Mice of both sexes (20–25 g, 4–5 months old) were used in this study. Animals were housed in groups of 6 mice in standard cages. *Ad libitum*, clean drinking water and food were provided. The mice were treated to a 12-hour light-dark schedule. Because mice are superior genetic models, researchers have increasingly used mice models rather than rats over the last two decades. Mice and humans have brains that have been evolutionarily conserved, which means that they have very similar brain architectures made up of similar types of brain cells.

### 2.3 Ethical

The Scientific Ethical Committee of Najran University, Saudi Arabia, approved the proposed work, and an ethical clearance certificate was obtained with reference number 443-41-29343-DS. The experiments were performed following international protocol for the humane handling of animals (NIH Publications No. 8023, revised 1978).

### 2.4 Chemicals

Pentylenetetrazole, Isoniazid, Diazepam, and Phenytoin were bought from Sigma Aldrich, United States of America. Assay Kits for biochemical parameters like GABA were purchased from Cayman's Chemical Company, USA. All the other reagents and chemicals were of standard analytical grade.

### 2.5 Collection and extraction of *V. indica* stem bark

The present study utilized the ethanolic extract of *V. indica* stem bark. The stem bark was collected from the western ghat forests of Dharwad, Karnataka, India. The bark was harvested in early spring season. The inner bark, which is often juicy, green, and aromatic, was harvested, since, the inner bark, not the outer bark, is the medicinal part of the bark. It was identified by a pharmacognosist, Dr Shastri Rajesh, Pharmacognosy Department, Faculty of Pharmacy, Soniya Education Trust, Dharwad, Karnataka, India, and a herbarium specimen is deposited with vide number: SETCPD/Ph.cog/herb/04/2017.

The collected bark was cleaned, split into short segments, shade-dried, crushed, and extracted with 100% ethanol using a Soxhlet apparatus for 12 h at 50 °C. The extract was evaporated using a rotary evaporator before being lyophilized into powder. The dried extract was dehydrated, and the yield was estimated in terms of dried plant material. After that, a suspension of the extract was made with 0.5 percent gum tragacanth and administered to the experimental animals.

### 2.6 Phytochemical evaluation and standardization

The VIE was investigated qualitatively for the presence of flavonoids, phenols, glycosides, tannins, alkaloids, saponins, steroids, and carbohydrates, among other phytochemicals. Furthermore, total phenols and total flavonoids content were quantified using the previously described procedure (Doss, 2009, Gupta et al., 2012). Mass Spectroscopy and FTIR were done as per standard procedures (Dharmender et al., 2010, Tine et al., 2017, Ayalew, 2020).

### 2.7 Acute toxicity study

The OECD-423 principles were followed for the acute toxicity investigation. Before dosing, the animals fasted for a night (OECD, 2002). VIE doses ranging from 5 mg/kg b.w. to 5000 mg/kg b.w. were given in a single dose via an oral feeding cannula. Each animal was monitored for the first 24 h following medication, with the first four hours receiving special attention, and then daily for the next 14 days. Hyperactivity, sedation, seizures, convulsions, hypothermia, grooming, and death were all observed toxicity factors. The selection of test doses for the current study was based on the results of the toxicity study. The extract was tolerated by the mice up to a level of 5000 mg/kg, hence we selected its one tenth part (500 mg/Kg p.o) as higher test dose; and one twentieth part (250 mg/kg p.o) as the lower test dose.

### 2.8 Experimental protocol

The mice were grouped randomly into the following groups (n = 6).

I) Determination of anticonvulsant activity
1. PTZ-induced convulsions:

Group I: Normal control (NC) was administered only vehicle (Normal saline).

Group II: Standard group (Diazepam 5 mg/kg p.o).

Group III: Positive control PTZ induced (PTZ 60 mg/ kg).

Groups IV: PTZ induced + VI Bark extract (low dose p.o).

Groups V: PTZ induced + VI Bark extract (high dose p.o).

2. Maximal electro shock (MES) induced convulsions:

Group I: NC (vehicle only).

Group II: Standard (Phenytoin 25 mg/kg p.o).

Group III: Positive control MES induced.

Group IV: MES induced + VI Bark extract (low dose p.o).

Group V: MES induced + VI Bark extract (high dose p.o).

3. Isoniazid (INH) induced convulsions:

Group I: NC (vehicle only).

Group II: Standard (Diazepam 5 mg/kg p.o).

Group III: Positive control INH induced (300 mg/kg).

Group IV: INH induced + VI Bark extract (low dose p.o).

Group V: INH induced + VI Bark extract (high dose p.o).

All the experimental models were performed as per standard procedures for Pentylenetetrazole (PTZ) induced convulsions (Koutroumanidou et al., 2013), Maximal electroshock (MES) induced convulsions (Swinyard et al., 1952), and Isoniazid (INH) induced convulsions (Bernasconi et al., 1985). On the last day of the experiments, mice were sacrificed by cervical dislocation followed by decapitation for isolation of the brains, and biochemical estimation and histopathology study of the brain tissue for all groups was carried out.

#### 2.8.1 Brain GABA estimation (Maynert et al., 1962)

Detached brains were homogenized in ice-cold 0.01 N HCl in a tube (5 ml). The homogenate was transferred directly to a tube containing cold 100% alcohol (8 ml) and kept at 0 °C for 1 h before centrifugation at 16,000 rpm for 10 min and collection in a Petri dish. The precipitate was rinsed three times with 75 percent alcohol (5 ml), and the supernatant was mixed with it. Evaporation on a water bath (70–90 °C) under a stream of air-dried the resulting supernatant. The dried product was dissolved in 2 ml chloroform plus 1 ml water, then centrifuged at 2000 rpm. The GABA-containing supernatant was collected, and 10 µl was used to make a spot on Whatman paper (No. 41).

#### 2.8.2 Chromatographic conditions

A paper chromatogram with an ascending method was utilized. For 30 min, the chromatographic chamber was saturated with n-butanol: acetic acid: water (50 ml: 12 ml: 60 ml) mobile phase. The Whatman paper was dried in a hot air oven before spraying a ninhydrin solution (0.5 percent in 95 percent ethanol) and dried for 60 min at 90 °C. The formed paper spot (blue colour) was sliced and placed in a beaker containing ninhydrin solution (2 ml), which was heated on a water bath for 5 min before adding 5 ml water and storing for 60 min. The resultant supernatant was collected, and the absorbance at 570 nm was measured.

#### 2.8.3 Standards and calculations

GABA (marketed) stock solution (1 mg/ml) was produced in 0.01 N HCl. Serial dilutions (1 ng/10 μl to 1000 ng/10 μl) were made from this stock solution. The preceding technique was repeated, except that the brain homogenate was replaced with commercially available GABA solutions to achieve the standard concentration curve for GABA.

#### 2.8.4 Histopathology

The isolated brain tissue was cut and immediately fixed in formalin solution (10%) for 24 h. The brain sections were dehydrated, fixed with paraffin, cut into 5 μm thick slices, and stained with hematoxylin and eosin dye. The prepared brain tissue sections were visualized under a light microscope (Kazmi et al., 2020).

### 2.9 *In-Vitro* antioxidant assay

#### 2.9.1 DPPH assay

Blois (1958) established a method for measuring test drugs' free radical scavenging activity (Blois, 1958). The test drug in multiple concentrations (20 to 200 µg/ml) were added to a 0.2 mM DPPH solution (100 μl). After 30 min, the absorbance was checked at 517 nm. Similarly, Ascorbic acid was used to plot a standard curve. Using the following equation, the scavenging activity was calculated:

Percentage of scavenging activity = (A0 − A1/A0) × 100.

A0: Control absorbance.

A1: Test absorbance.

#### 2.9.2 Reducing power assay

The previously mentioned standard method was used to estimate reducing power activity (Yildirim et al., 2001). The test drug in multiple concentrations (50 to 450 µg/ml) were introduced to a test tube containing 1 ml of 200 mM sodium phosphate buffer (pH 6.6) and 1 ml of 1 percent potassium ferricyanide solution and incubated for 20 min at 50 °C. One milliliter of 10% trichloroacetic acid was added to it, and centrifuged for 10 min (3000 rpm). To the subsequent supernatant ferric chloride (0.5 ml) and distilled water (2 ml), was added and incubated for 10 min before the absorbance was measured (700 nm). Greater absorbance indicates that the test compounds have a more substantial reductive power (Jamuna et al., 2012).

#### 2.9.3 Hydroxyl radical scavenging activity

The ^•^OH scavenging activity was determined using the deoxyribose protocol (Halliwell et al., 2006). 10 mM 2-deoxyribose (0.15 ml), 0.2 M Na3PO4 buffer (0.45 ml), 10 mM H2O2 (0.15 ml), 10 mM FeSo4-EDTA (0.15 ml), distilled water (0.525 ml), and test drug (0.075 ml) were added to the reaction mixture and incubated for 4 h at 37 °C. After incubation, 2.8% trichloroacetic acid (0.75 ml) and 1.0% thiobarbituric acid (0.75 ml) were added, and the mixture was simmered for 10 min before being cooled and the absorbance measured at 520 nm. For comparison, mannitol (0.5–4.5 mg/ml) was employed as a standard, and a standard curve was constructed.

Percentage of ^•^OH radical scavenging = (A0 − A1/A0) × 100.

A0: Control absorbance.

A1: Test absorbance.

#### 2.9.4 Statistical analysis

The data were presented in the form of mean SEM. One-way ANOVA was used to analyze statistical differences, followed by the Post-Tukeys test. P < 0.05 was judged statistically significant for all comparisons. Graph-Pad Prism software was used for all statistical analyses.

## 3 Results

### 3.1 Phytochemical evaluation

The presence of flavonoids, phenolics, glycosides, tannins, polysaccharides, saponins, and steroids was confirmed by qualitative analysis in the ethanol extract of *V. indica* bark. The quantitative estimation revealed total phenol and flavonoid concentrations of 580.96 mg GAE/g extract and 66.89 mg RE/g extract, respectively.

### 3.2 FTIR and Mass spectroscopy

**Fig. 1:**
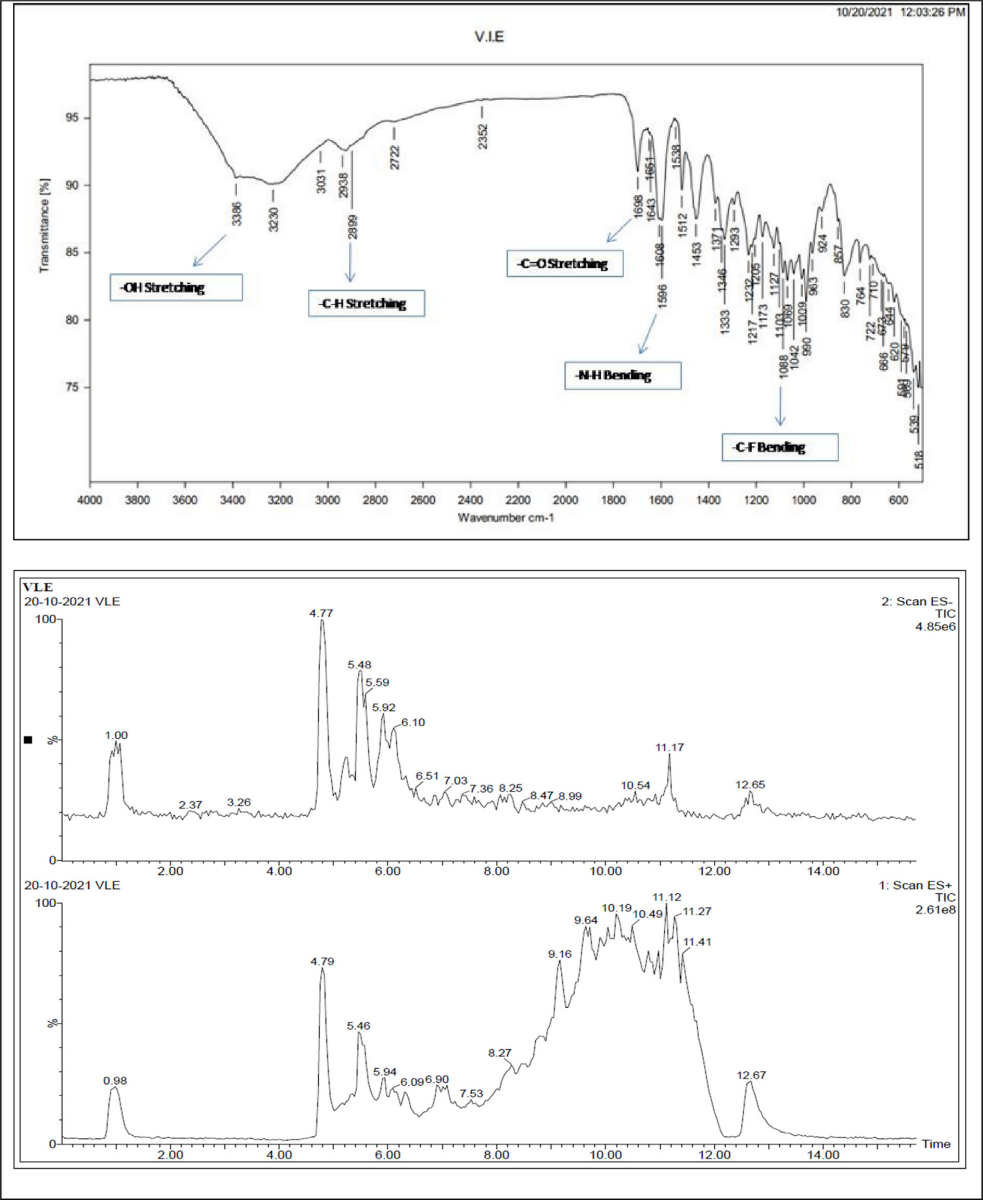
FTIR and Mass spectra of *Vateria indica* ethanolic extract.

The spectral analysis data confirmed several compounds by searching NIST/EPA/NIH Mass Spectral Library (NIST 17) mass spectral library in ChemData.NIST.GOV. The spectral analysis results corroborate with previous reports (Fig. 1). The list of compounds confirmed by MS are depicted in Table 1.

**Table 1:** Mass Spectra of *Vateria indica* confirming the presence of several phytochemicals.

S. No	**Compound Name**	**Reported Mol.Wt**	**Obtained Mol.Wt**
	Vaticanol C	906.9	906.96
	Epsilon Viniferin	454.97	454.47
	Stilbenes	180.25	179.28
	Vaterioside A	614.603	613.35
	Bergenin	328.27	328.98
	Vaticanol B	906.9	906.10
	Resveratrol	220.35	219.25
	Veterioside E	614.603	613.35
	Isocumarin	146.14	147.23
	Viniferin	454.47	453.08
	Viniferol	680.7	678.39
	Kaempferol-3-O-rhamnoside	432.4	432.73
	Kaempferol-3-O-rhamnopyranosyl	740.7	741.30
	Quercitrin	448.4	447.89
	Diterpenes (Columbin)	358.4	358.91
	Diterpenes (Palmarin)	374.4	374.67
	L-Fisetinidol	274.26	275.18
	Vaticanol B	906.9	906.10

#### 3.2.1 Acute toxicity study

The extract was tolerated by the mice up to a level of 5000 mg/kg. They all survived the test, and there were no noticeable changes in their appearance or general behaviour.

#### 3.2.2 VIE protects mice from convulsions caused by maximal electrical shock (MES)

**Fig. 2:**
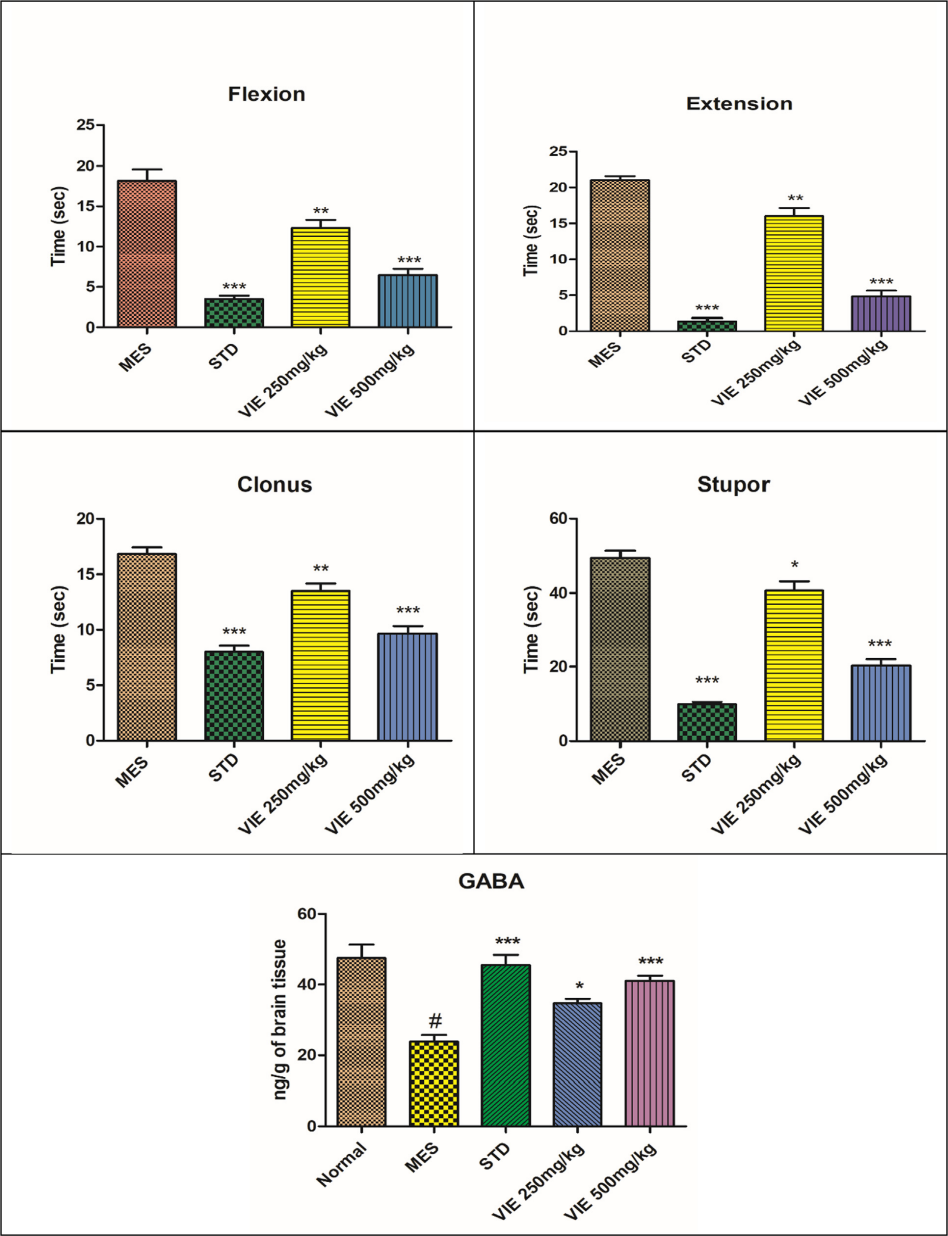
Effect of VIE on MES-induced convulsions and GABA. Values are expressed as Mean ± SEM for 6 animals per group.*P < 0.05; **P < 0.01; ***P < 0.001 compared with positive control; **#** P < 0.001 compared to normal control.

VIE significantly decreased all convulsive phases caused by MES (tonic flexion phase, tonic extensor phase, clonic convulsion phase, and stupor) compared to MES control. Likewise, mice given phenytoin (25 mg/kg) were protected from MES-induced convulsions, as evidenced by the lack of all convulsion stages. Fifty percent of animals recovered in the MES control group, while treatment with phenytoin provided significant recovery (100%) to all mice. At the same time, two-thirds of mice treated with VIE showed recovery against MES (Table 2 and Fig. 2).

**Table 2:** Effect of VIE on maximal electroshock (MES) induced convulsions.

**Groups**	**Tonic Flexion (s)**	**Tonic hind limb extension: THLE (sec)**	**Clonic Convulsion (sec)**	**Stupor (sec)**	**Recovery/Death**
MES control	18.17 ± 1.42	21 ± 0.57	16.83 ± 0.6	49.33 ± 2.01	50% recovered
STD Phenytoin 25 mg/kg	3.5 ± 0.42***	1.33 ± 0.49***	8 ± 0.57***	9.83 ± 0.6***	All recovered
VIE 250 mg/kg	12.33 ± 0.98**	16 ± 1.15**	13.5 ± 0.67**	40.67 ± 2.48*	66.66% recovered
VIE 500 mg/kg	6.5 ± 0.76***	4.83 ± 0.79***	9.66 ± 0.66***	20.33 ± 1.76***	83.33% recovered

Values are expressed as Mean ± SEM for 6 animals per group.

*P < 0.05; **P < 0.01; ***P < 0.001 compared with controls (ANOVA followed by post hoc tests for multiple comparisons).

#### 3.2.3 VIE protects against INH- induced convulsions in mice

**Fig. 3:**
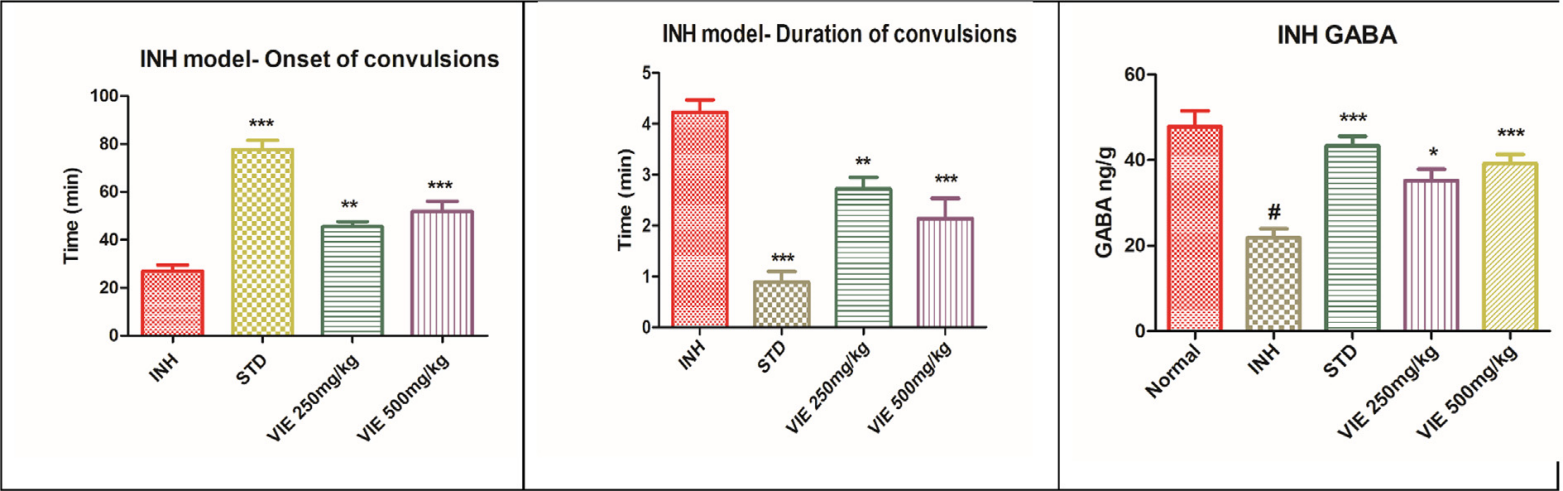
Effect of VIE on INH-induced seizure activity and GABA levels in mice. Values are expressed as Mean ± SEM for 6 animals per group.*P < 0.05; **P < 0.01; ***P < 0.001 compared with positive control; **#** P < 0.001 compared to normal control.

As depicted in Table 3, administration of INH (300 mg/kg) produced convulsions and death in all the experimental animals in the positive control group (mortality rate 100%). However, VIE intervention showed significant protection (P < 0.001) against INH induced seizure activity and reduced the mortality rate up to 16.6% compared to INH alone group (mortality rate 100%). Furthermore, VIE treatment significantly delayed the onset of seizure episodes and reduced the duration of seizure activity. Similarly, Diazepam (standard drug) treatment substantially reduced seizure occurrences and provided complete protection since none of the animals died following INH treatment in this group. (Fig. 3).

**Table 3:** Effect of VIE on Isoniazid-induced seizure episodes.

**Groups**	**No. of animals showing convulsions**	**Animals protected (%)**	**Mortality (%)**	**Latency to Clonic convulsions (minutes)**	**Duration of Clonic convulsions (minutes)**
Positive control INH (300 mg/kg)	6/6	0%	6/6 (100%)	26.98 ± 2.46	4.227 ± 0.24
INH + Diazepam 5 mg/kg i.p.	0/6	100%	0/6 (0%)	77.75 ± 3.8 ***	0.885 ± 0.21***
INH + VIE 250 mg/kg p.o.	2/6	66.6%	2/6 (33.3%)	45.45 ± 2.17 **	2.718 ± 0.22**
INH + VIE 500 mg/kg p.o.	1/6	83.3%	1/6 (16.6%)	51.91 ± 4.18 ***	2.135 ± 0.39***

Values are expressed as Mean ± SEM for 6 animals per group. **P < 0.01; ***P < 0.001compared with controls (ANOVA followed by post hoc tests for multiple comparisons).

#### 3.2.4 VIE protects against PTZ- induced convulsions

**Fig. 4:**
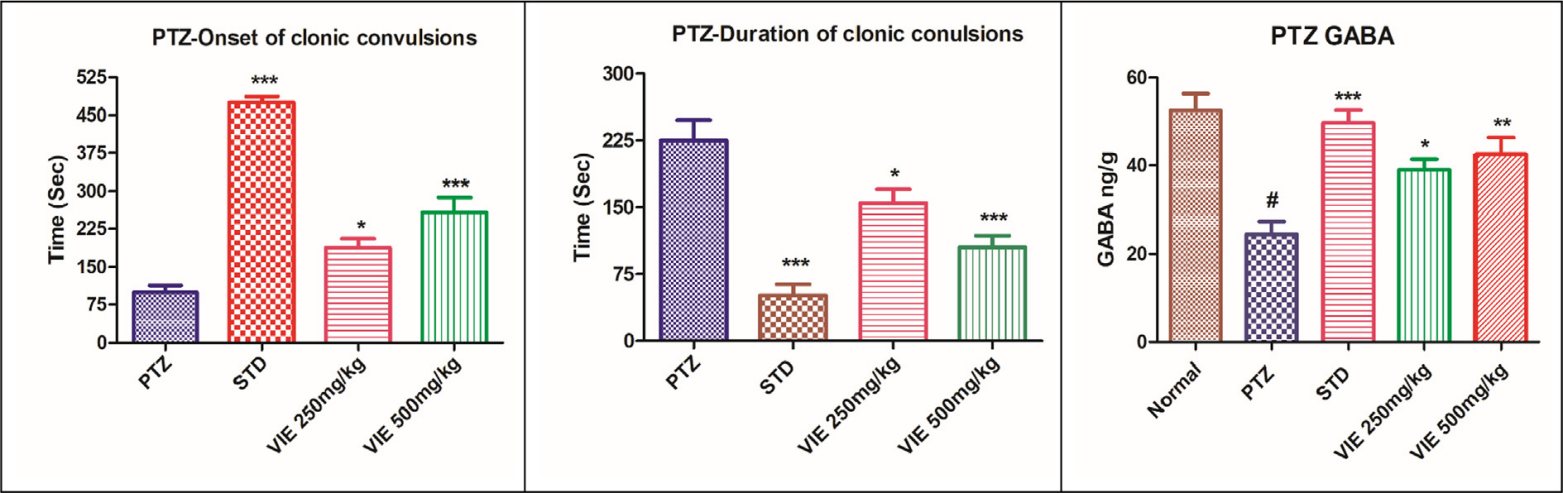
Effect of VIE on PTZ-induced seizure activity and GABA. Values are expressed as Mean ± SEM for 6 animals per group.*P < 0.05; **P < 0.01; ***P < 0.001 compared with positive control; **#** P < 0.001 compared to normal control.

PTZ elicited hind- and fore limb myoclonic twitches in all animals 60–70 s after i.p. administration. This was proceeded by a collapse with one side or the back, which was followed by generalized clonic discharges. In a dose-dependent way, VIE pretreatment considerably delayed the latency to clonic convulsions (Table 4). Moreover, VIE intervention demonstrated a significant reduction in the intensity and duration of clonic convulsions. In addition, pretreatment with VIE showed protection against the PTZ-induced seizures and reduced the mortality rate (Fig. 4).

**Table 4:** Effect of VIE on PTZ-induced seizure episodes.

**Groups**	**No. of animals showing convulsions**	**Animals protected (%)**	**Mortality (%)**	**Latency to Clonic convulsions (Seconds)**	**Duration of Clonic convulsions (Seconds)**
Positive control PTZ (80 mg/kg, i.p)	6/6	0%	6/6 (100%)	99.30 ± 13.98	225.0 ± 22.91
PTZ + Diazepam 5 mg/kg i.p.	0/6	100%	0/6 (0%)	474.3 ± 11.97 ***	51 ± 12.73 ***
PTZ + VIE 250 mg/kg p.o.	3/6	50%	3/6 (50%)	186.8 ± 18.24 **	154.6 ± 15.71 **
PTZ + VIE 500 mg/kg p.o.	2/6	66.6%	2/6 (33.4%)	257.4 ± 19.73 ***	105.2 ± 12.89 ***

Values are expressed as Mean ± SEM for 6 animals per group. **P < 0.01; ***P < 0.001compared with controls (ANOVA followed by post hoc tests for multiple comparisons).

### 3.3 VIE restored the brain GABA content

PTZ, INH, and MES administration significantly reduced brain GABA levels in positive control mice compared to normal control animals. VIE intervention dose-dependently restored brain GABA levels (Table 5).

**Table 5:** Effect of VIE on GABA levels in mice brains.

**Treatment Groups**	**GABA levels (ng/g of brain tissue)**		
	**MES**	**INH**	**PTZ**
Normal control	47.5 ± 3.8	47.83 ± 3.7	52.50 ± 3.81
Positive control	23.83 ± 1.9 **^#^**	21.83 ± 2.12 **^#^**	24.50 ± 2.93 **^#^**
Phenytoin 25 mg/kg i.p.	45.5 ± 2.93 ***	–	–
Diazepam 5 mg/kg i.p.	–	43.33 ± 2.24 ***	49.67 ± 2.90 ***
VIE 250 mg/kg p.o	34.67 ± 1.3 *	35.17 ± 2.7 *	39 ± 2.44 *
VIE 500 mg/kg p.o	41 ± 1.52 ***	39.17 ± 2.18 ***	42.50 ± 3.81**

Values are Mean ± SD, n = 6 in each group. **P < 0.01,***P < 0.001 when compared with positive control groups. **#** P < 0.001 compared to normal control.

#### 3.3.1 VIE incurs protection against oxidative stress

***Free radical scavenging activity (DPPH***^•^***):*** VIE's DPPH^•^ scavenging action was concentration-dependent, increasing linearly from 20 to 200 µg mL^−1^. VIE scavenging activity peaked at 200 µg mL^−1^ (86.36% inhibition), and standard antioxidant ascorbic acid scavenging activity peaked at 200 µg mL^−1^ (95.40% inhibition). VIE and ascorbic acid showed IC50 values of 96.91 µg/ml and 68.13 µg/ml, respectively.

***Reducing power assay:*** The reduction power of VIE was dose-dependent, and at a concentration of 500 µg mL^−1^, VIE had the most significant reducing power. VIE and ascorbic acid had IC50 values of 419.16 µg/ml and 374.24 µg/ml, respectively.

***Hydroxyl radical scavenging activity:*** VIE prevented deoxyribose decomposition, with 500 µg mL^−1^ exhibiting the maximum hydroxyl scavenging activity. VIE and mannitol IC50 values were determined to be 293.75 µg mL^−1^ and 175.3 µg mL^−^^1^, respectively.

#### 3.3.2 Histopathology

**Fig. 5:**
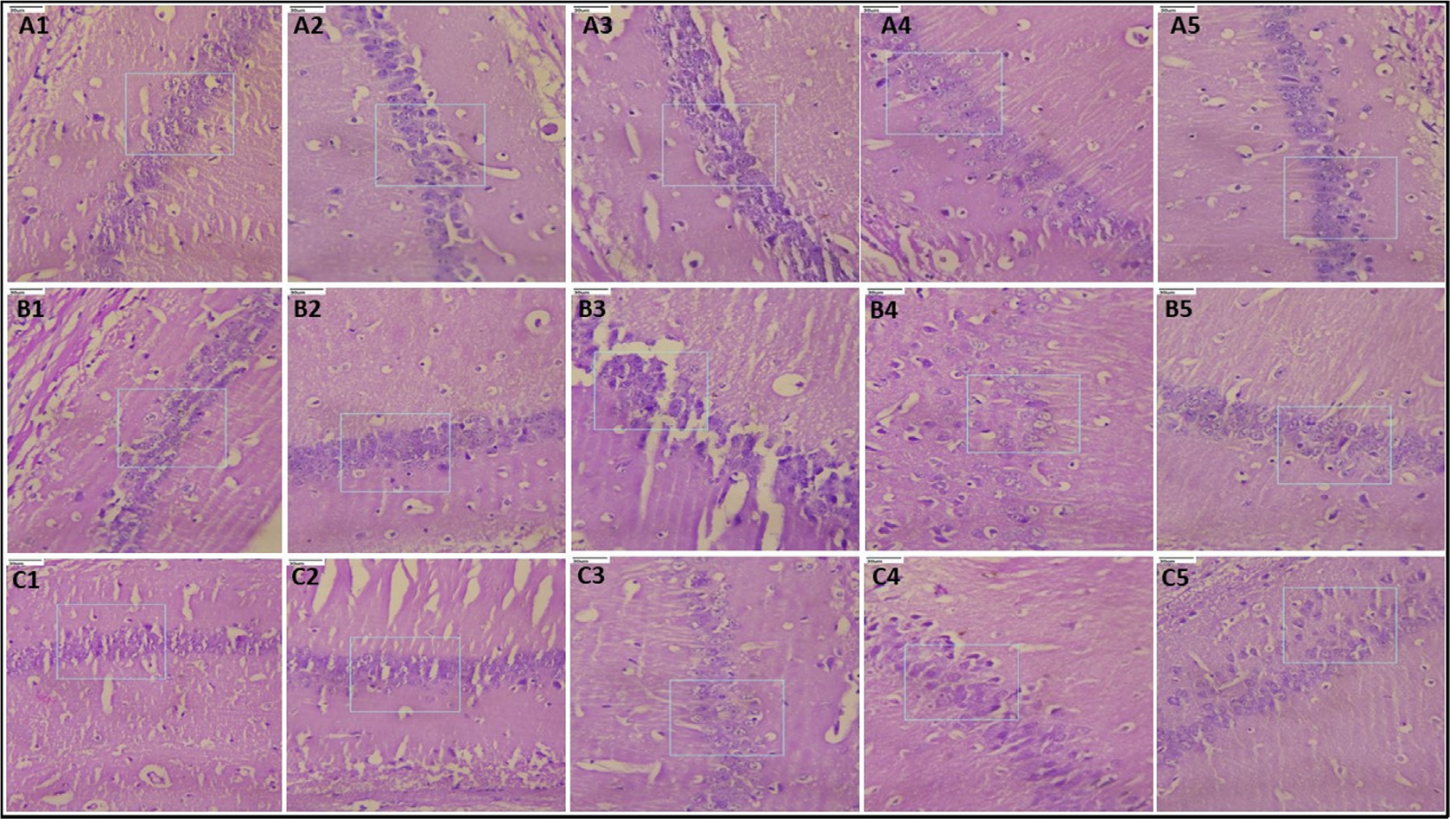
Photomicrographs of mice brain tissue (40x) in PTZ-model (A-series); INH-model (B-series); MES-model (C-series). 1) Normal control; 2) Positive control; 3) Standard; 4) VIE 250 mg/Kg; 5) VIE 500 mg/Kg.

Fig. 5 depicts the photomicrographs of the brain sections of mice. Normal control animal brains showed normal neuronal cells without cerebral congestion or edema. Brain tissue from the positive control group showed marked cerebral congestion, cerebral edema, meningeal congestion and neuronal eosinophilia. Brain tissue in animals treated with standard drugs and VIE 500 mg/Kg did not reveal cerebral congestion, cerebral edema, meningeal congestion, neuronal eosinophilia and meningeal inflammation. Brain from animals treated with VIE 250 mg/kg exhibited relatively less cerebral congestion, cerebral edema, meningeal congestion and meningeal inflammation.

## 4 Discussion

Epilepsy is a collection of syndromes marked by repeated spontaneously occurring seizures that appear to be caused by complicated mechanisms involving numerous neurotransmitter pathways in the brain, including GABAergic, cholinergic, and glutamatergic systems (De Almeida et al., 2011). Epilepsy affects approximately 50 million individuals worldwide, making it one of the most prevalent neurodegenerative disorders, affecting an estimated 1.5% of the world population (Beghi, 2020). For epilepsy management, numerous antiseizure medications are being used. They do, however, have dangerous side effects like ischemia, depression, motor disability, and impaired cognition. In addition, conventional medications are synthetic, resulting in severe adverse effects and drug dependence.

Furthermore, approximately 30% of epilepsy patients are refractory to treatment with the antiepileptic drugs that are presently available.

Herbal drugs derived from folkloric medicine have played an essential role in developing modern medications. They can serve as a source of antiepileptic drugs with unique structures and improved efficacy and safety profiles. In the context of ongoing drug discovery for novel antiepileptic lead candidates, the current study seeks to analyze the prospective antiepileptic and antioxidant capabilities of *Vateria indica* and its influence on the brain's GABAergic system.

In the present study, we used the MES, INH, and PTZ models for screening antiepileptic drugs. The application of electrical stimuli in the MES model generated considerable seizure activity in the MES-control group, as evidenced by increased clonic convulsions, tonic hind limb extension, tonic flexion, and stupor in the positive control group. In addition, the GABA levels in the MES-control group were low compared to normal control animals, and all animals (100%) in the MES-control group had seizure activity, with a 50% death rate.

As evidenced by a decline in the duration of all stages of MES-induced seizures, as well as elevated brain GABA levels, VIE intervention considerably reduced MES-induced convulsions. Furthermore, VIE intervention rendered protection to all the animals, as none of the animals died. These results are consistent with Alkahtani et al. (2021), who reported that treating mice with *Garcinia mangostana* significantly reversed the MES-induced convulsions and had significant antiepileptic potential modulated through positive GABAergic neurotransmission and antioxidant properties.

Moreover, in the INH and PTZ-induced seizure models, VIE significantly delayed clonic convulsions' latency (min) and lowered clonic convulsions' duration (min). When compared to positive-control mice, VIE exhibited a dose-dependent beneficial effect on GABAergic neurotransmission, as evidenced by a substantial rise in brain GABA levels. The VIE intervention demonstrated a significant neuroprotective effect on the treated mice, parallel to the protective effect exhibited by the standard drug diazepam. These findings are consistent with recent research that reported the therapeutic potential of herbal medicines to be protective against INH, PTZ, and MES-induced convulsion models (Ahmed Alkahtani, 2021, Gupta et al., 2014, Sun et al., 2019, Vijayalakshmi et al., 2011). For the study of absence seizures and generalized myoclonic siezures, the PTZ model is appropriate. On the other hand, the MES and INH-animal models are good predictors of generalized tonic-clonic seizure treatment efficacy.

It is well established that in several animal models (INH, PTZ, and MES models) employed to screen antiepileptic agents, a decrease in GABAergic neurotransmission is responsible for the epileptogenic actions. The main inhibitory neurotransmitter in the brain is GABA, and increasing GABA neurotransmission has been shown to mitigate convulsions (Vijayalakshmi et al., 2011). PTZ is thought to work at the molecular level through noncompetitive antagonism of the GABA_A_ receptor complex (Hansen et al., 2004). It has been suggested that medications that suppress T-type Ca2 + currents, such as ethosuximide, and compounds that promote GABA_A_ receptor-mediated inhibitory neurotransmission, such as benzodiazepines and phenobarbital, can prevent PTZ-induced seizures (De Sarro et al., 1999). In addition, INH's epileptogenic activity is linked to disruption in GABAergic neurotransmission by blocking the vital enzyme glutamic acid decarboxylase, which is responsible for GABA production from glutamic acid (Drug Discovery and Evaluation, 2014).

Furthermore, the pathophysiology of epilepsy has been linked to neuronal hyperexcitability and the excessive generation of free radicals. The brain is susceptible to free radical damage due to its high rate of oxidative metabolism, limited antioxidant defences, and high polyunsaturated fatty acid content (Devi et al., 2008). Both oxidative and nitrosative stress are thought to play a role in the pathogenesis of epilepsy. Several studies (animal models and genetic studies) have shown that persistent seizures cause an increase in mitochondrial oxidative stress and subsequent cell damage (Waldbaum et al., 2010, Liang and Patel, 2006, Gluck et al., 2000).

Consequently, antioxidant agents that aim to minimize oxidative stress have garnered considerable interest in the treatment modality of epilepsy (Devi et al., 2008). In the current study, the phytochemical investigation revealed a high content of total phenols and flavonoid concentrations in the VIE extract. Thus, any compound which acts by countering the oxidative stress is considered to be an effective treatment modality in the management of epileptic disorders, as demonstrated in the current study. In addition, histology of brain tissue showed that animals treated with VIE did not reveal cerebral congestion, cerebral edema, meningeal congestion, neuronal eosinophilia, or meningeal inflammation compared to positive-control animals. Thus, VIE's ability to improve GABAergic neurotransmission as well as its antioxidant potential, as evidenced by its DPPH- and hydroxyl radical scavenging ability and reducing power activity, can be linked to its seizure-protective action.

## 5 Conclusion

To conclude, *Vateria indica* may provide adequate protection against epileptic seizures, suggesting that it could be used to treat petitmal and grandmal epilepsy. Our findings are preliminary, and more research is needed before animal data can be applied to humans. We plan to provide pure lead compounds derived from *Vateria indica* in the future in order to better understand the role it could play in the development of natural anticonvulsant therapies.

## Funding

The Deanship of Scientific Research of Najran University in Najran, Saudi Arabia, funded this research vide grant no. NU/-/MRC/10/383.

## Declaration of Competing Interest

The authors declare that they have no known competing financial interests or personal relationships that could have appeared to influence the work reported in this paper.

## References

[b0005] Abdel-Wahab B.A., Shaikh I.A., Khateeb M.M., Habeeb S.M. (2015). Omega 3 polyunsaturated fatty acids enhance the protective effect of levetiracetam against seizures, cognitive impairment and hippocampal oxidative DNA damage in young kindled rats. Pharmacol. Biochem. Behav..

[b0010] Ahmed Alkahtani S. (2021). Role of Gamma-Aminobutyric Acid in the Antiepileptic Activity of Garcinia Mangostana Standardized Extract. Curr. Top Nutraceutical Res..

[b0015] Al Rajeh S., Awada A., Bademosi O., Ogunniyi A. (2001). The prevalence of epilepsy and other seizure disorders in an Arab population: A community-based study. Seizure.

[b0020] Alshabi A.M., Shaikh I.A., Savant C. (2020). Nootropic and neuroprotective effects of ethanol extract of *Vateria indica* L bark on scopolamine-induced cognitive deficit in mice. Trop. J. Pharm. Res..

[b0025] Ayalew A.A. (2020). Chromatographic and spectroscopic determination of solvent-extracted Lantana camara leaf oil. J. Int. Med. Res..

[b0030] Beghi E. (2020). The Epidemiology of Epilepsy. Neuroepidemiology..

[b0035] Bernasconi R., Klein M., Martin P. (1985). The specific protective effect of diazepam and valproate against isoniazid-induced seizures is not correlated with increased GABA levels. J. Neural Transm..

[b0040] Blois M.S. (1958). Antioxidant determinations by the use of a stable free radical [10]. Nature.

[b0045] Choudhary N., Bijjem K.R.V., Kalia A.N. (2011). Antiepileptic potential of flavonoids fraction from the leaves of Anisomeles malabarica. J. Ethnopharmacol..

[b0050] Corda M.G., Giorgi O., Orlandi M., Longoni B., Biggio G. (1990). Chronic administration of negative modulators produces chemical kindling and GABAA receptor down-regulation. Adv. Biochem. Psychopharmacol..

[b0055] De Almeida R.N., De Fátima A.M., Maior F.N.S., De Sousa D.P. (2011). Essential oils and their constituents: Anticonvulsant activity. Molecules.

[b0060] De Sarro A., Cecchetti V., Fravolini V., Naccari F., Tabarrini O., De Sarro G. (1999). Effects of novel 6-desfluoroquinolones and classic quinolones on pentylenetetrazole-induced seizures in mice. Antimicrob. Agents Chemother..

[b0065] Devi P.U., Manocha A., Vohora D. (2008). Seizures, antiepileptics, antioxidants and oxidative stress: An insight for researchers. Expert Opin. Pharmacother..

[b0070] Dharmender R., Madhavi T., Reena A., Sheetal A. (2010). Simultaneous Quantification of Bergenin, (+)-Catechin, Gallicin and Gallic acid; and Quantification of β-Sitosterol using HPTLC from Bergenia ciliata (Haw.) Sternb. Forma ligulata Yeo (Pasanbheda). Pharm. Anal. Acta.

[b0075] Doss A. (2009). Preliminary phytochemical screening of some Indian Medicinal Plants. Anc Sci. Life.

[b0080] Drug Discovery and Evaluation: Pharmacological Assays, 2014. doi: 10.1007/978-3-642-27728-3.

[b0085] French J.A., Williamson P.D., Thadani V.M., Darcey T.M., Mattson R.H., Spencer S.S., Spencer D.D. (1993). Characteristics of medial temporal lobe epilepsy: I. Results of history and physical examination. Ann. Neurol..

[b0090] Gupta G., Afzal M., David S., Verma R., Candaswamy M., Anwar F. (2014). Anticonvulsant activity of Morus alba and its effect on brain gamma-Aminobutyric acid level in rats. Pharmacognosy Res..

[b0095] Gupta N., Lobo R., Chandrashekhar K.S., Gupta D. (2012). Determination of phenol and flavonoid content from *Vateria indica* (Linn). Der Pharm Lett..

[b0100] Gluck M.R., Jayatilleke E., Shaw S., Rowan A.J., Haroutunian V. (2000). CNS oxidative stress associated with the kainic acid rodent model of experimental epilepsy. Epilepsy Res..

[b0105] Halliwell B., Grootveld M., Gutteridge J.M.C. (2006). Methods for the measurement of hydroxyl radicals in biochemical systems: Deoxyribose degradation and aromatic hydroxylation. Methods Biochem. Anal..

[b0110] Hansen S.L., Sperling B.B., Sánchez C. (2004). Anticonvulsant and antiepileptogenic effects of GABAA receptor ligands in pentylenetetrazole-kindled mice. Prog. Neuro-Psychopharmacol. Biol. Psychiatry..

[b0115] Jamuna S., Paulsamy S., Karthika K. (2012). Screening of in vitro antioxidant activity of methanolic leaf and root extracts of hypochaeris radicata L (Asteraceae). J. Appl. Pharm. Sci..

[b0120] Kazmi Z., Zeeshan S., Khan A., Malik S., Shehzad A., Seo E.K., Khan S. (2020). Antiepileptic activity of daidzin in PTZ-induced mice model by targeting oxidative stress and BDNF/VEGF signaling. Neurotoxicology.

[b0125] Koutroumanidou E., Kimbaris A., Kortsaris A., Bezirtzoglou E., Polissiou M., Charalabopoulos K., Pagonopoulou O. (2013). Increased Seizure Latency and Decreased Severity of Pentylenetetrazol-Induced Seizures in Mice after Essential Oil Administration. Epilepsy Res. Treat..

[b0130] Lévesque M., Avoli M. (2013). The kainic acid model of temporal lobe epilepsy. Neurosci. Biobehav. Rev..

[b0135] Liang L.-P., Patel M. (2006). Seizure-induced changes in mitochondrial redox status. Free Radic. Biol. Med..

[b0140] Lopes M.W., Lopes S.C., Costa A.P. (2015). Region-specific alterations of AMPA receptor phosphorylation and signalling pathways in the pilocarpine model of epilepsy. Neurochem. Int..

[b0145] Magaji M.G., Yaro A.H., Musa A.M., Anuka J.A., Abdu-Aguye I., Hussaini I.M. (2012). Central depressant activity of butanol fraction of Securinega virosa root bark in mice. J. Ethnopharmacol..

[b0150] Makkar R., Behl T., Bungau S., Zengin G., Mehta V., Kumar A., Uddin M.S., Ashraf G.M., Abdel-Daim M.M., Arora S., Oancea R. (2020). Nutraceuticals in neurological disorders. Int. J. Mol. Sci..

[b0155] Maynert E.W., Klingman G.I., Kaji H.K. (1962). Tolerance to morphine. II. Lack of effects on brain 5-hydroxytryptamine and gamma-aminobutyric acid. J. Pharmacol. Exp. Ther..

[b0160] Meldrum B.S. (1999). Antiepileptic drugs potentiating GABA. Electroencephalogr. Clin. Neurophysiol. Suppl..

[b0165] OECD, 2002. Test No. 423: Acute Oral toxicity - Acute Toxic Class Method. Oecd Guidel Test Chem. December, 1–14. doi: 10.1787/9789264071001-en.

[b0170] Pithadia A.B., Navale A., Mansuri J., Shetty R.S., Panchal S., Goswami S. (2013). Reversal of experimentally induced seizure activity in mice by glibenclamide. Ann. Neurosci..

[b0175] Quintans J.S.S., Antoniolli Â.R., Almeida J.R.G.S., Santana-Filho V.J., Quintans-Júnior L.J. (2014). Natural products evaluated in neuropathic pain models - a systematic review. Basic Clin. Pharmacol. Toxicol..

[b0180] Spagnuolo C., Napolitano M., Tedesco I., Moccia S., Milito A., Luigi R.G. (2016). Neuroprotective Role of Natural Polyphenols. Curr. Top. Med. Chem..

[b0185] Sun Z., Meng F., Tu L., Sun L. (2019). Myricetin attenuates the severity of seizures and neuroapoptosis in pentylenetetrazole kindled mice by regulating the of BDNF-TrkB signaling pathway and modulating matrix metalloproteinase-9 and GABAA. Exp. Ther. Med..

[b0190] Swinyard E.A., Brown W.C., Goodman L.S. (1952). Comparative assays of antiepileptic drugs in mice and rats. J. Pharmacol. Exp. Ther..

[b0195] Tine Y., Renucci F., Costa J., Wélé A., Paolini J. (2017). A method for LC-MS/MS profiling of coumarins in zanthoxylum zanthoxyloides (Lam.) B. Zepernich and timler extracts and essential oils. Molecules.

[b0200] Turski W.A., Cavalheiro E.A., Schwarz M., Czuczwar S.J., Kleinrok Z., Turski L. (1983). Limbic seizures produced by pilocarpine in rats: Behavioural, electroencephalographic and neuropathological study. Behav. Brain Res..

[b0205] van der Burg K.P., Cribb L., Firth J., Karmacoska D., Sarris J. (2021). Nutrient and genetic biomarkers of nutraceutical treatment response in mood and psychotic disorders: a systematic review. Nutr. Neurosci..

[b0210] Vijayalakshmi A., Ravichandiran V., Anbu J., Velraj M., Jayakumari S. (2011). Anticonvulsant and neurotoxicity profile of the rhizome of Smilax china Linn. in mice. Indian J. Pharmacol..

[b0215] Waldbaum S., Liang L.-P., Patel M. (2010). Persistent impairment of mitochondrial and tissue redox status during lithium-pilocarpine-induced epileptogenesis: Mitochondrial oxidative stress in pilocarpine-induced epilepsy. J. Neurochem..

[b0220] Walia K.S., Khan E.A., Ko D.H., Raza S.S., Khan Y.N. (2004). Side Effects of Anti-epileptics- A Review. Pain Pract..

[b0225] Yildirim A., Mavi A., Kara A.A. (2001). Determination of antioxidant and antimicrobial activities of Rumex crispus L. extracts. J. Agric. Food Chem..

